# Sex and menopause-based differences in presentation of early Lyme disease: A prospective cohort study

**DOI:** 10.1007/s10238-026-02063-0

**Published:** 2026-02-07

**Authors:** Alison W. Rebman, Ting Yang, John N. Aucott

**Affiliations:** Lyme Disease Research Center, Division of Rheumatology, Department of Medicine, Johns Hopkins University School of Medicine, 2360 W Joppa Rd. Suite 320, Lutherville-Timonium, Baltimore, MD 21093 USA

**Keywords:** Lyme disease, Sex, Serologic tests, Symptom reporting, Diagnosis

## Abstract

Although prior research has established sex and menopausal status-based differences in immune response, susceptibility, and severity to a variety of pathogens, their relevance in early Lyme disease is understudied. We examined the clinical and serologic presentation of patients with early Lyme disease, stratified first by sex then by menopausal status. We also explored the hypothesis that males would present with more severe early Lyme disease. In this prospective cohort study from the Mid-Atlantic US, 243 adult, antibiotic-naïve patients were enrolled with a diagnostic erythema migrans rash present. Demographic, physical exam, symptom, laboratory, and two-tier serology data were collected at a baseline, and a post-treatment visit 3 weeks later. Lyme disease severity was operationalized through six indicators: rash size, number of acute symptoms, dermatologic dissemination, positive serology, liver function elevation, and elevated neutrophil-lymphocyte ratio. Unadjusted group comparisons and multivariate regression adjusting for potential confounders were used to assess difference. In logistic models adjusted for age, Lyme disease duration, systemic steroid use, and co-morbid thyroid disease, males had higher odds of testing two-tier positive (OR = 1.77 [1.03, 3.04], *p* = 0.039). This difference was more pronounced between males and pre-menopausal females (OR = 2.93 [1.26–6.79], *p* = 0.012) and no significant difference was found comparing males to post-menopausal females. In ordinal logistic models with Lyme disease severity as the outcome adjusted for age and Lyme disease duration, males had higher odds of being in a higher disease severity score category (OR = 1.94 [1.20,3.15], *p* = 0.028); again, particularly in comparison to pre-menopausal females (OR = 2.26 [1.13,4.58], *p* = 0.044). Heart palpitations (*p* = 0.023), vomiting (*p* = 0.007), and photophobia (*p* = 0.057) trended towards higher reporting among females, while sleep difficulty (*p* = 0.010) was higher among males. No differences were found on non-dermatologic components of the physical exam. We found sex and menopausal status to be relevant in accounting for variability in two-tier serologic status and severity of early Lyme disease in a well-characterized group of patients. Lower rates of seroreactivity among females is unexpected but may be consistent with lower acute severity of disease. Our clinical findings underscore the need for additional research to understand possible contributing biologic and/or social behavioral factors, as well as their impact on timely diagnosis and post-treatment conditions.

**Supplementary Information:**

The online version contains supplementary material available at 10.1007/s10238-026-02063-0.

## Abstract (plain language summary)

Lyme disease is a bacterial infection obtained through a tick bite. The goal of this study was to look at whether male and female patients with early Lyme disease show up to the doctor with different signs of their disease in terms of the symptoms they report, their physical exams, and the results of their laboratory tests. We also examined whether females who had gone through menopause would be different on these factors compared to those who had not. We studied data from 243 adults (118 females and 125 males) with early Lyme disease before and after treatment. We found that at diagnosis, males were more likely to have a positive test and more obvious findings of severe disease, yet there were no differences in how long males and females had been sick. For both of these findings, the male group was more similar to females who had undergone menopause and was more different than females who had not. We found a small number of Lyme disease symptoms that were reported more frequently among females (heart palpitations, vomiting, eyes sensitive to light, neck pain, nausea) and two symptoms (sleep difficulty and irritability) reported more frequently among males. These findings suggest that sex and menopause status are important to consider in understanding early Lyme disease. More research is needed to determine the cause of these differences and their impact on time to diagnosis and risk of later conditions after treatment.

**Supplementary Information:**

The online version contains supplementary material available at 10.1007/s10238-026-02063-0.

## Highlights

Male sex was associated with significantly higher odds of two-tier antibody positivity in early Lyme disease.

Severity of early Lyme disease, based on objective markers of disease impact, was higher among males.

When stratified by menopausal status, post-menopausal females appeared more similar to males in disease severity and two-tier antibody reactivity than pre-menopausal females.

The overall number of symptoms reported was not associated with patient sex, however heart palpitations and vomiting were significantly more common among females and sleep difficulty was significantly more commonly reported among males.

**Supplementary Information:**

The online version contains supplementary material available at 10.1007/s10238-026-02063-0.

## Background

Lyme disease (LD) is a tick-borne infection caused by genospecies of the spirochete bacteria *B. burgdorferi* sensu lato [1]. In the US, cases are most frequently reported in areas of the Northeast, Mid-Atlantic, Upper Midwest, and Pacific Coast states [2]. Early LD is characterized by the presence of the erythema migrans (EM) skin lesion, non-specific infection-type symptoms such as fever, myalgia, and fatigue, and/or antibodies to *B. burgdorferi*. These antibodies can take days or weeks to develop, frequently producing negative test results in early infection [1, 3]. In later stages, the untreated infection can progress to cardiac, neurologic, or musculoskeletal system involvement. Following appropriate antibiotic treatment, a subset of patients will continue to report persistent symptoms of fatigue, pain, cognitive difficulty, sleep disruption, and others [4]. 

Significant prior research has established that males and females differ both in their susceptibility to and severity of a variety of different infectious diseases. This includes a wide range of pathogenic viruses, bacteria, and parasites [5–7]. The cause of this difference is complex. Hormonal, genetic, and environmental effects all likely contribute to sex-based immunologic differences in response to infection and subsequent clinical outcomes [8]. Moreover, changes in sex hormones during and after the female menopause transition have also been shown to be associated with alterations in the immune system [9–11]. Across a number of contagions, males are generally considered to be at higher risk of more severe initial infection, whereas females may be at increased risk from post-acute immunologic perturbations [7, 12]. 

Despite increasing recognition in recent decades of sex-based differences in health, their relevance in the clinical presentation of LD has been understudied, and menopausal status has rarely been mentioned or examined in this context. The aim of this study was to explore the clinical and serologic presentation of antibiotic-naïve patients with early LD, stratified first by sex and then by menopausal status among females. First, we specifically examined symptom profiles, physical exam findings, and two-tier serologic positivity among males and females, adjusting for potential confounders to ensure robustness of findings. Then, we operationalized and explored the hypothesis that males present with more severe early LD, as this has not previously been examined.

## Methods

### Study participants

Adult participants from the Mid-Atlantic US were enrolled in a longitudinal, prospective cohort study (the *S*tudy of *L*yme disease *I*mmunology and *C*linical *E*vents [*SLICE*]), as previously described [13]. Participants either self-referred or were recruited from primary or urgent care settings in 2008–2024. Participants were required to have a physician-documented EM ≥ 5 cm diagnostic for early LD. As the original study was designed with post-treatment persistent symptoms as the outcome, participants were excluded for a range of self-reported, co-morbid conditions including myalgic encephalomyelitis/chronic fatigue syndrome (ME/CFS), fibromyalgia, unexplained chronic pain, sleep apnea or narcolepsy, autoimmune disease, chronic neurologic disease, liver disease, HIV, cancer or malignancy in the past 2 years, major psychiatric illness, or drug or alcohol abuse.

Participants were followed for 5 visits over 1 year in this study; however, the current analyses drew only from data collected at the baseline acute (V1) and subsequent convalescent visit approximately 3 weeks later at the end of antibiotic treatment (V2). In these analyses, we also excluded patients who had started antibiotic treatment at V1, and those who reported a prior episode of LD. This was done to; (a) avoid potential confounding of symptom reporting by antibiotic side effects (either resolution or worsening in the initial days of treatment), and (b) avoid the potential effects of previous LD episodes on patients’ current clinical and serologic presentation.

The Institutional Review Board of the Johns Hopkins University School of Medicine approved the studies from which these data were drawn, and no study activities were performed prior to obtaining written, informed consent.

### Variables of interest

All participants self-reported sex as ‘male’, ‘female’, or ‘other’ at V1. Menopausal status was defined by self-report of no menstrual period for one year or surgical removal of ovaries. Menopausal status was also verified against patients’ self-reported sex, age, and date of last menstrual period (if reported) to assess for internal consistency.

At V1, participants also completed interviewer-administered questionnaires regarding their LD. To assess symptoms during acute infection, participants were asked at V1 if they had experienced any of 36 symptoms as part of their current illness. A write-in option was also provided to ensure that we captured all significant symptoms. Participants were specifically prompted to only endorse symptoms that were new, different, or significantly worse than their normal since the onset of LD. These symptoms could begin shortly before, or more commonly after, first noticing the EM rash [14]. A standard physical exam was also performed at V1 which included evaluation and measurement of the EM. Finally, participants underwent a standard clinical complete blood count (CBC) and complete metabolic panel (CMP), the results of which were used to assess group differences in liver function tests (LFT) and to calculate a neutrophil to lymphocyte (NLR) ratio.

Standard two-tier serologic testing (STTT) for antibodies to *B. burgdorferi* was performed at V1 and V2. Interpretation to positive or negative at each visit followed CDC guidance which incorporates estimated duration of illness at the time of testing [15]. Results were analyzed by sex and menopausal status in two ways. First, patients were categorized both as positive at V1, and as ‘ever positive’, a composite of either testing positive at V1 or V2. Second, we also displayed results on individual test components (ELISA, IgM, and IgG) at V1 and V2. In our initial study protocol (48.6% of participants), clinical two-tier guidance was followed wherein a reflex Western blot (WB; IgM and IgG) was only performed subsequent to a positive ELISA, and participants who were ELISA and WB positive at V1 were not retested at V2. This protocol was later revised to perform complete ELISA and WB testing at both visits for research purposes (51.4% of participants). Therefore, not all participants had complete ELISA, IgM, and IgG data available at both time points.

### Statistical methods

For continuous variables, two group (male vs. female, and pre-vs. post-menopausal) unadjusted comparisons were performed using Wilcoxon rank sum test, while three group (male vs. pre-menopausal female vs. post-menopausal female) unadjusted comparisons were performed using Kruskal-Wallis test. Chi square or Fisher’s exact test were used for all unadjusted group comparisons of categorical variables. Logistic regression was used to compare the odds of ‘ever positive’ (as the dependent variable) across all pairwise sex/menopausal groups (primary independent variables): male vs. female, male vs. pre-menopausal female, male vs. post-menopausal female, and post- vs. pre-menopausal female. Possible confounding factors were selected based on their potential to be independently associated with both sex/menopausal status and serologic positivity, while not acting as mediators or colliders.

We operationalized severity of early disease by first creating binary indicators of the following six variables; EM size, number of acute symptoms, disseminated EM, ever two-tier positive, ≥ 1 elevated LFT, and NLR ratio. Continuous variables were dichotomized to above or below the overall sample median (EM size: median = 80 cm^2^; number of acute symptoms: median = 6; NLR ratio: median = 2.97). A radar plot was used to display the percentage of more severe acute disease across males, pre-menopausal females, and post-menopausal females for each indicator. A severity score was then created by summing these binary disease indicators (total: 0–6). Finally, a series of ordinal logistic regression (proportional odds) models were fitted to compare differences in this severity score (as the dependent variable) across all pairwise sex/menopausal groups (primary independent variables): male vs. female, post- vs. pre-menopausal female, male vs. pre-menopausal female, and male vs. post-menopausal female. The proportional odds assumption which assumes that the effect of each covariate is consistent across all levels of the score was formally evaluated using the Brant test. Model results are expressed as odds ratios (OR) with 95% confidence intervals. Models comparing male and female groups were adjusted for age and illness duration, whereas only illness duration was adjusted for in the comparison between post- and pre-menopausal females due to collinearity between age and menopausal status and subsequent challenges in interpretation.

In all adjusted analyses, individual variables were first evaluated using univariate models. Factors showing potential significant association with the dependent variable (*p* < 0.15) were then included in a final multivariate model. The *p*-values resulting from separate models were corrected for multiple comparisons using the Benjamini-Hochberg procedure to control the false discovery rate (FDR).

A *p*-value less than 0.05 was considered statistically significant for all analyses. To assess the stability and robustness of the observed association between sex/menopausal status and outcomes, we performed Bootstrap Inclusion Frequency (BIF) analyses. We generated 1,000 bootstrap resamples with replacement from the original dataset. For each resample, we refit the proper multivariable regression model with potential confounders kept fixed in the model and recorded the frequency with which the primary predictor remained a statistically significant predictor (p-value < 0.05). The BIF represents the percentage of bootstrap samples in which the predictor was significant, with higher percentages indicating greater stability of the inference. In the BIF analyses, the significance of the predictor sex/menopausal status was determined using a nominal significance threshold of *p* < 0.05 to assess the individual structural stability of each model, while primary manuscript *p*-values remain FDR-adjusted to account for multiple testing. All analyses were performed using SAS version 9.4 (SAS Institute, Cary, NC) or R version 4.4.1 (R Foundation for Statistical Computing, Vienna, Austria). Graphs were generated using Prism version 10.2.2 (GraphPad Software, San Diego, CA) or BioRender Software (Toronto, Ontario).

## Results

### Demographic and clinical comparisons

A total of 407 participants with early LD were enrolled. We removed 159 (39.1%) who had taken at least one dose of antibiotic treatment at V1, four (0.98%) with a prior history of LD, and one (0.25%) missing serology tests at both V1 and V2 from the analyses, resulting in a final sample of 243 participants.

Table 1 shows the overall as well as the sex- and menopausal status-stratified demographic and clinical characteristics of this sample. There were similar proportions of males (51.44%) and females (48.56%), and among females, a majority (66.9%) were post-menopausal. Female participants were on average 9 years older than males. No patients in our sample were diagnosed with disseminated neurologic or cardiac LD at enrollment, although two female post-menopausal patients developed VII nerve palsy between V1 and V2. There were no differences across groups in estimated LD duration at V1 with an overall median of 6 days. As expected, thyroid disease was significantly more common among females compared to males, and heart disease was more common among post-menopausal compared to pre-menopausal females.

**Table 1:** Demographic and clinical characteristics by sex and menopausal status among a sample of 243 antibiotic-naïve patients with early Lyme disease from the Mid-Atlantic US.^1^

	All *N* = 243	Male *N* = 125	Female			*p* Male vs. Female	*p* Pre- vs. Post-Menopause
			All *N* = 118	Pre-Menopause *N* = 39	Post-Menopause *N* = 79		
Age (Years)	53.0 (40.0–62.0) [20.0–84.0]	47.0 (35.0–59.0) [20.0–76.0]	56.0 (47.0–64.0) [20–84.0]	41.0 (30.0–49.0) [20.0–56.0]	62.0 (56.0–68.0) [38.0–84.0]	0.0004	< 0.0001
White Black Asian Other/>1 Race	231 (95.06%) 1 (0.41%) 3 (1.23%) 8 (3.29%)	120 (96.0%) 0 (0.00%) 2 (1.60%) 3 (2.40%)	111 (94.07%) 1 (0.85%) 1 (0.85%) 5 (4.24%)	33 (84.62%) 1 (2.56%) 1 (2.56%) 4 (10.26%)	78 (98.73%) 0 (0.00%) 0 (0.00%) 1 (1.27%)	0.574	0.007
Hispanic Ethnicity	3 (1.23%)	1 (0.80%)	2 (1.69%)	1 (2.56%)	1 (1.27%)	0.613	1.000
Education (Years)^2^	16.0 (16.0–18.0) [9.0–26.0]	16.0 (16.0–18.0) [9.0–26.0]	17.0 (16.0–19.0) [11.0–23.0]	16.0 (16.0–19.0) [14.0–23.0]	17.0 (16.0–19.0) [11.0–23.0]	0.069	0.960
Estimated Disease Duration (Days)	6.0 (4.0–10.0) [1.0–70.0]	5.0 (4.0–10.0) [1.0–70.0]	6.0 (4.0–12.0) [1.0–70.0]	7.0 (3.0–10.0) [1.0–70.0]	6.0 (4.0–14.0) [1.0–60.0]	0.276	0.498
Disseminated Erythema Migrans	74 (30.45%)	42 (33.60%)	32 (27.12%)	8 (20.51%)	24 (30.38%)	0.273	0.257
Erythema Migrans Size (cm^2^)^3^	79.0 (35.0–161.0) [3.63–1296.0]	90.0 (42.58–198.0) [3.63–1296.0]	56.0 (28.0–130.0) [4.83–640.0]	55.0 (28.0) [130.0-14.52.0.52-289.0]	56.88 (28.0–130.0) [4.83–640.0]	0.0007	0.908
Number of Acute Symptoms	6.0 (2.0–11.0) [0.0–30.0]	6.0 (2.0–11.0) [0.0–30.0]	6.0 (2.0–12.0) [0.0–22.0]	5.0 (1.0–13.0) [0.0–22.0]	6.0 (2.0–11.0) [0.0–22.0]	0.894	0.950
≥ 1 Elevated Liver Function Test^4^	68/238 (28.57%)	38/122 (31.15%)	30/116 (25.86%)	4 (10.26%)	26/77 (33.77%)	0.367	0.006
Neutrophil-to-Lymphocyte Ratio	2.96 (2.04–4.47) [0.74–33.93]	3.25 (2.31–4.47) [0.74–14.05]	2.49 (1.90–4.48) [0.81–33.93]	2.49 (1.94–4.07) [1.40–6.99]	2.48 (1.89–5.03) [0.81–33.93]	0.057	0.862
Systemic Corticosteroids in Prior 3 Months	7 (2.88%)	2 (1.60%)	5 (4.24%)	1 (2.56%)	4 (5.06%)	0.270	1.000
Diabetes	6 (2.47%)	3 (2.40%)	3 (2.54%)	0 (0.00%)	3 (3.80%)	1.000	0.550
Thyroid Disease	26 (10.70%)	1 (0.80%)	25 (21.19%)	4 (10.26%)	21 (26.58%)	< 0.0001	0.041
Heart Disease or Hypertension	35 (14.40%)	19 (15.20%)	16 (13.56%)	1 (2.56%)	15 (18.99%)	0.716	0.014

^1^Normally distributed continuous variables are presented as mean (standard deviation) and non-normally distributed variables are presented as median (inter-quartile range) [range]

^2^Three males, one pre-menopausal female, and one post-menopausal female missing years of education

^3^One post-menopausal female missing erythema migrans size

^4^Defined as aspartate aminotransferase > 35, alanine aminotransferase > 40, or alkaline phosphatase > 130 for males and > 115 for females. Three males and 2 post-menopausal females missing liver function testing

We have previously reported sex-based differences in EM size and NLR abnormalities, and these were also found in this cohort comprised of many of the same patients (Table 1) [13, 16]. The only clinical difference found by menopausal status was in the proportion with an elevated LFT, with post-menopausal females having almost three times the rate as pre-menopausal females. In stratified univariate logistic regression models, a 10-year increase in age was associated with significantly higher odds of abnormal LFT among females (OR: 1.48 [1.06, 2.15], Wald χ² = 4.83, df = 1, *p* = 0.028) but did not reach significance among males (OR: 1.25 [0.96, 1.65], Wald χ² = 2.68, df = 1, *p* = 0.101). However, a formal test of homogeneity using an interaction term (age X sex) confirmed that the effect of age did not differ significantly by sex (males vs. females: ratio of odds ratios [ROR]: 0.85 [0.54, 1.31], Wald χ² = 0.55, df = 1, *p* = 0.459).

On physical exam, similar proportions of males and females were found to have lymphadenopathy (12.8% male vs. 10.2% female, *p* = 0.521), a present liver span (male 4.0% vs. female 5.1%, *p* = 0.684), and a present spleen tip (3.2% male vs. 0.0% female, *p* = 0.123). Among females, none of these physical exam findings varied significantly by menopausal status (*p* > 0.662 for each).

### Symptom presentation

**Fig. 1:**
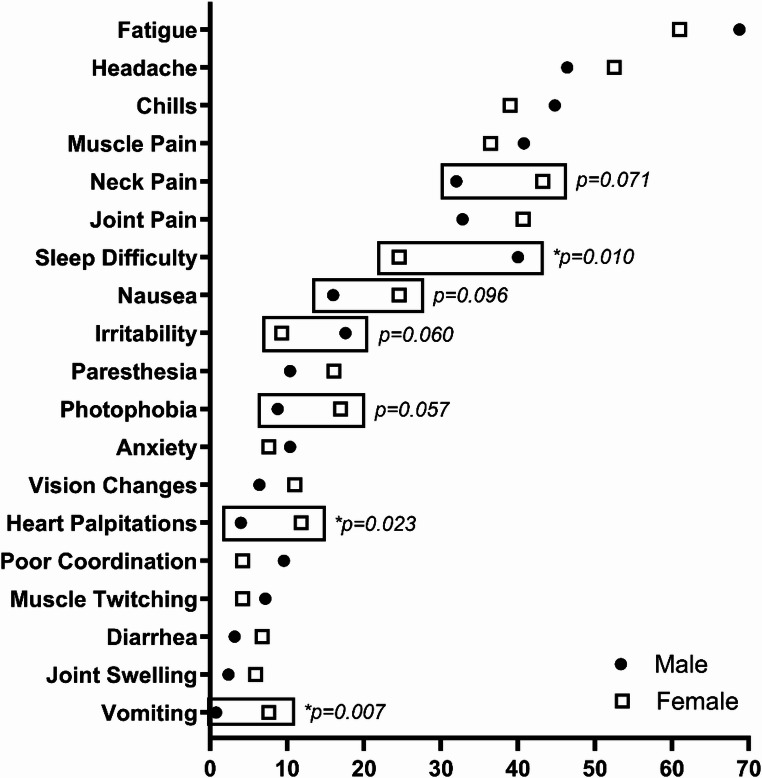
Early Lyme disease symptoms (percent reporting) among antibiotic-naïve males (*n* = 125) and females (*n* = 118). Symptoms are displayed if (a) they were present in > 2.5% of the sample as a whole, or (b) if there was a > 2.5% difference in frequency between males and females. Symptoms are ordered by their overall prevalence in the sample. Symptoms which were statistically significantly different by sex (*p* < 0.05, indicated with*) or trending towards statistical significance (0.05 < *p* < 0.10) are indicated by a box. Drooping eyelids, double vision, facial weakness, skin sensitivity, eye pain, stiffness, and nerve pain are not shown for low frequency in the sample as a whole (present in ≤ 2.5%). Urination changes, memory changes, sore throat, back pain, fever, breathing difficulty, difficulty finding words, dizziness, tinnitus, sweats, depression, loss of appetite, swollen lymph nodes, and difficulty focusing or concentrating are not shown for a lack of difference between males and females (≤ 2.5% difference in percentage between males and females)

There was no significant difference between males and females, or between pre-and post-menopausal females, in the total number of acute symptoms reported (Table 1). Among the 39 individual symptoms examined, only 3 (7.7%) were statistically significantly endorsed by different proportions of males and females (Fig. 1). Vomiting and heart palpitations were significantly more frequently reported among females, while sleep difficulty was significantly more frequently reported among males. Photophobia, neck pain, and nausea were also reported more frequently among females, while irritability was reported more frequently among males, although these did not reach statistical significance (0.05 < *p* < 0.10). Among females, no acute disease symptoms were statistically significantly different by menopausal status, and only sore throat showed a statistical trend (18.0% pre- vs. 6.3% post-menopausal, *p* = 0.060).

**Fig. 2:**
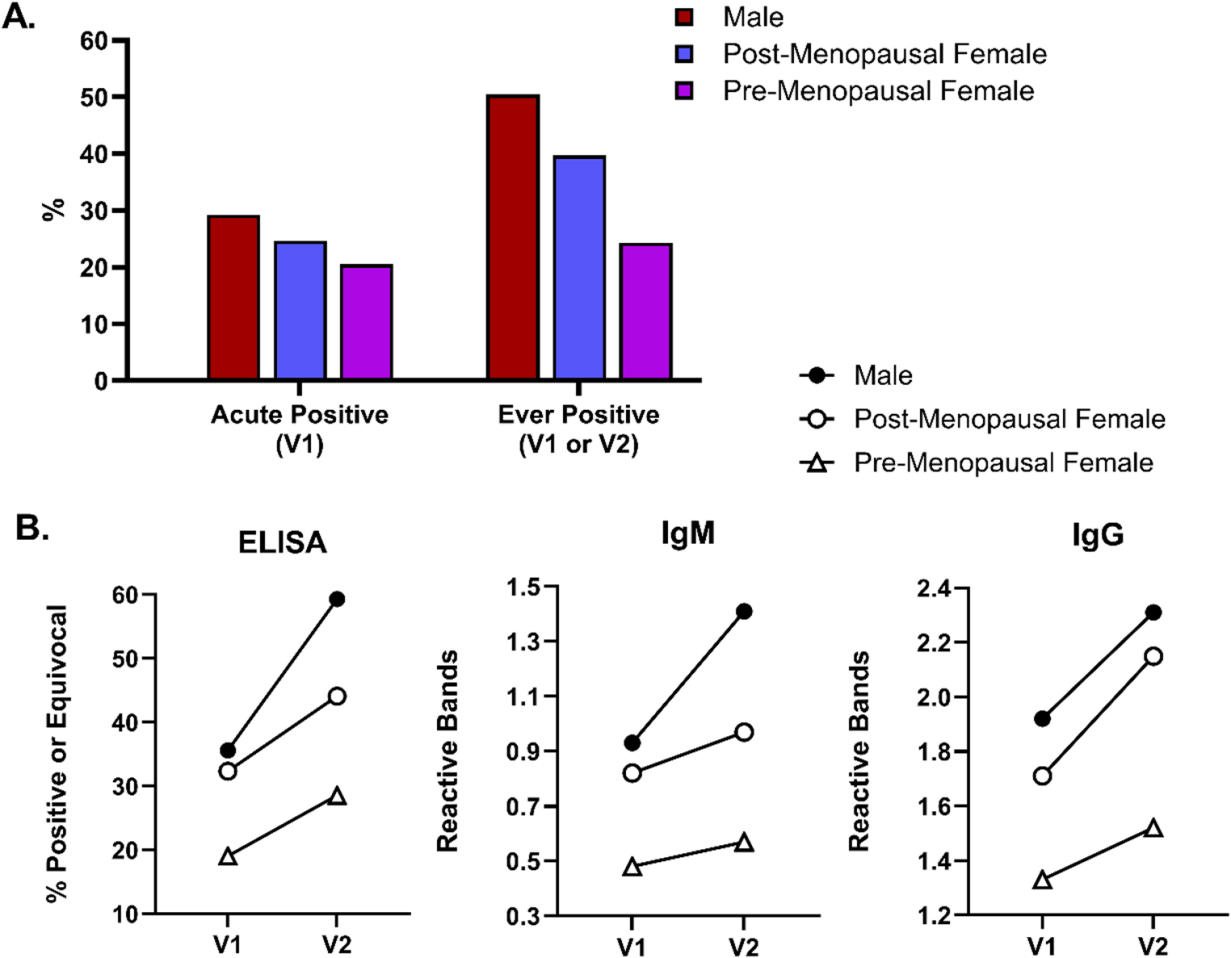
Two-tier serology testing by sex and menopausal status among 243 antibiotic naïve participants with early Lyme disease.**A.** The proportion two-tier positive at the acute (V1), and combined (V1/V2) time points. Four patients (1.6%) were missing results at V1, and 14 patients (5.8%) were missing final combined results. **B.** The proportion ELISA positive, and the number of reactive IgM and IgG at the acute (V1) and three-week convalescent (V2) time points. Only 114 participants with complete ELISA, IgM, and IgG data at both time points were included to avoid potential bias introduced by missing data. The sex and menopausal status distribution of the 114 included participants was not statistically significantly different than the 129 not included (*p* = 0.540). To account for changes in laboratory-determined upper limit values over the course of the study, ELISA is displayed as a binary (negative vs. equivocal/positive) rather than a continuous variable

### Two-Tier serologic testing

Figure 2A shows the proportion of patients who were positive at V1 and the proportion who were ‘ever positive’, stratified by sex and menopausal status. When the test is broken down into individual components (ELISA, IgM, and IgG), differences by sex and menopausal status were also descriptively present (Fig. 2B). Across all three components, pre-menopausal females appeared lower at V1, and as a group, did not increase as notably during the 3-week treatment interval between V1 and V2.

We then sought to test if these group differences remained in adjusted models with ‘ever seropositive’ as the dependent variable. We selected the following as potential confounders; age, duration of illness, recent systemic steroid use, co-morbid thyroid disease, and V2 test requirement (whether IgG was required at this later time point or IgM alone was sufficient, based on estimated duration of illness at V2). Table 2 shows the results of unadjusted and adjusted models with sex as the primary independent variable of interest. In univariate analyses, only the IgM/IgG variable was identified as having a confounding effect and included in the final model. We found that males had higher odds of testing positive than females (OR = 1.77 [1.03,3.04], Wald χ² = 4.25, df = 1, *p* = 0.039, BIF = 58.3%) (Table 2).

**Table 2:** Unadjusted and adjusted logistic regression models examining the association between serologic positivity and sex

	Unadjusted		Adjusted	
	Odds Ratio [95% CI]	*p*-value	Odds Ratio [95% CI]	*p*-value
Male Sex (vs. Female)	1.93 [1.13, 3.28]	0.029	1.77 [1.03, 3.04]	0.039
V2 Required IgG (vs. IgM or IgG)	0.54 [0.32, 0.91]	0.034	0.59 [0.34, 1.01]	0.055
Age (per 10 years)	1.04 [0.87, 1.24]	0.653		
Duration of illness (per week)	1.05 [0.87, 1.27]	0.651		
Systemic Steroids in past 3 months	0.53 [0.10, 2.76]	0.546		
Thyroid Disease	0.68 [0.29, 1.59]	0.513		

In similar adjusted models with sex/menopausal status included as the primary independent variable instead, we observed that males had almost three times the odds of testing positive compared to pre-menopausal females (OR = 2.93 [1.26,6.79], Wald χ² = 6.29, df = 1, *p* = 0.012, BIF = 75.9%) (Supplemental Table 2 A) but no significant difference was found compared to post-menopausal females (OR = 1.41 [0.77, 2.57], Wald χ² =1.24, df = 1, *p* = 0.266) (Supplemental Table 2B).

### Clinical severity

**Fig. 3:**
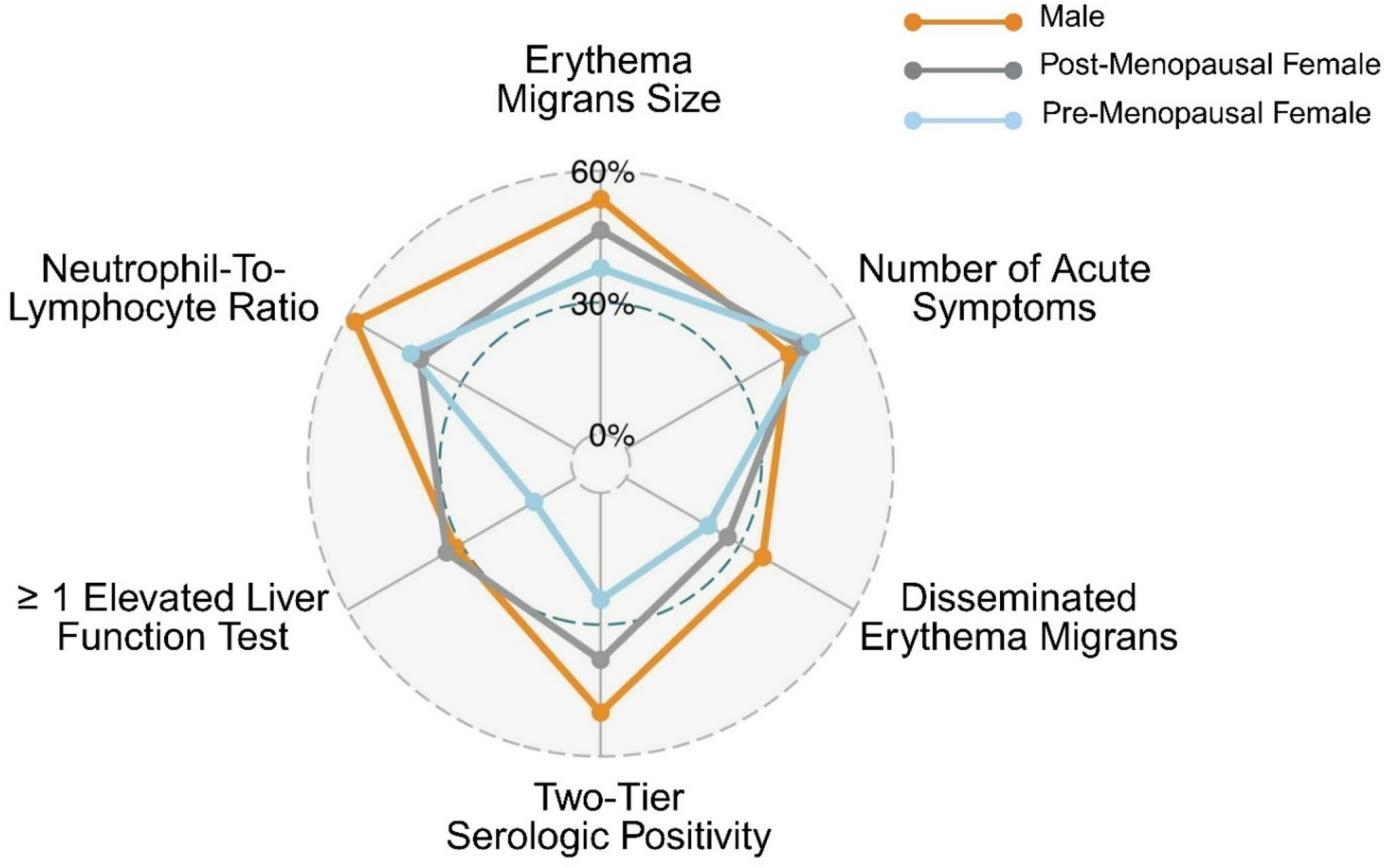
Radar plot of early Lyme disease characteristics by sex and menopausal status among 239 antibiotic-naïve patients. For each characteristic, the percentage abnormal (for binary variables) or the percentage above the sample median (for continuous variables) is plotted on the graph

Figure 3 depicts the frequency of specific acute disease indicators by sex and menopausal status. In three-group comparisons, apart from number of symptoms, males exhibited a trend towards higher frequencies of more severe acute disease characteristics than pre- or post-menopausal females, including significant group differences in two-tier serologic positivity, ≥ 1 elevated liver function test, and neutrophil-to-lymphocyte ratio (*p* ≤ 0.052 for each).

A disease composite score was generated as the sum of all six nodes in Fig. 3. Ordinal logistic regression (proportional odds) models were found to be appropriate for the composite score analysis, as the proportional odds assumption was fully met (Brant test p-values ranging from 0.37 to 0.74). In models adjusted for age and duration of illness, male sex was associated with a 94% increase in the odds of being in a higher severity score category compared to females (OR = 1.94 [1.20,3.15], Wald χ² = 7.27, df = 1, *p* = 0.028, BIF = 79.3%) (Supplemental Table 3). In similar adjusted models with sex/menopausal status as the primary predictor, males exhibited significantly higher odds of being in a higher severity score category than pre-menopausal females (OR = 2.26 [1.13,4.58], Wald χ² = 5.23, df = 1, *p* = 0.044, BIF = 62%). A non-significant trend was observed when comparing males to post-menopausal females (OR = 1.68 [0.93,3.04], Wald χ² = 2.90, df = 1, *p* = 0.118) (Supplemental Table 3).

## Discussion

Prior epidemiological studies have consistently shown a slight majority male in early LD case surveillance reporting from high-incidence states, while males are even more significantly over-represented in later stages of untreated LD infection such as carditis and arthritis [17–20]. Conversely, females appear to be at increased risk of persistent, post-treatment LD symptoms [21], consistent with their over-representation across other post-acute infection syndromes such as long COVID or ME/CFS [22, 23]. These trends suggest sex-based differences in disease risk, clinical presentation, and/or diagnosis, although potential mechanisms remain unknown and largely unexplored. In this study, we examined the clinical and serologic presentation of antibiotic-naïve, early LD by sex and menopausal status among patients from the Mid-Atlantic US presenting with EM rash at the time of diagnosis. We found sex and menopause status to be relevant factors in accounting for patient variability in two-tier serology and severity of acute disease in our cohort. Despite an overall similar symptom burden by sex, we also found subtle differences in a small number of the symptoms reported. Together, these findings inform both a scientific understanding of the host response in early LD as well as the practical, enduring clinical challenge of early diagnosis.

In many bacterial and viral infections such as sepsis and SARS-COV-2, females have been shown to be less susceptible to more severe disease than males [7, 24]. This is thought to arise from generally stronger innate and adaptive immune responses, particularly for pre-menopausal females [5, 11, 25]. These include stronger Th1-type responses and expression of higher levels of pro-inflammatory cytokines, rendering females at immunologic advantage in fighting infection. Hospitalization rates are low in early LD [26] and mortality is very rare, therefore disease severity in this setting represents a narrower range of clinical presentations compared to other infections such as SARS-COV-2. To test the hypothesis that a similar trend would be observed and males would present with more severe disease, we operationalized ‘severity’ based on objective markers of disease impact commonly identifiable in the clinical setting of early LD diagnosis: EM size, number of acute symptoms, *B. burgdorferi* dissemination with multiple skin lesions, and abnormal laboratory indicators (positive antibody test, and elevated LFT or NLR). Some of the differences we observed in these individual factors have been reported previously in subsets of this cohort [13, 16]. In the current study, with the exception of number of symptoms, we descriptively found a similar trend in all markers, and our overall disease severity composite score was significantly higher for males in adjusted models.

It is seemingly unexpected that males in our sample would have significantly higher odds of two-tier serologic reactivity. Ostensibly, the reverse might be anticipated as females generally have stronger immune responses and documented higher antibody responses to vaccines [27]. However, higher antibody reactivity among males was observed in our previous, small chart review sample [28] and in a more recent publication by Lit et al. based on national testing data [29]. In convalescent plasma donors following SARS-COV-2 infection, male sex was associated with both increased disease severity and increased antibody responses [30]. In that case, the authors suggest that more severe disease in males could heighten inflammatory responses and B-cell recruitment, leading to more antibody production. We are not the first to suggest a similar biological mechanism for observed sex-based differences in LD. Strle et al. discussed the possibility of greater spirochetemia among males, perhaps resulting from differences in immunologic response to infection [19]. We hypothesize a similar process as a possible explanation for our findings, as higher rates of seroconversion, particularly in convalescent testing, have been found to be associated with spirochetemia [31] and clinical factors such as disseminated EM [32]. This is supported by models showing higher spirochete load among male mice [33, 34], and the consistent over-representation of males across data sources in later, disseminated stages of untreated LD such as carditis and arthritis [17–20]. 

It is also possible that socio-behavioral factors may contribute to the findings we observed. In a recent study of national sex-specific LD testing trends in over 1 million individuals, male odds of a positive test were also found to be over 2 times those of females, similar to our results [29]. The authors suggest socio-behavioral explanations for their findings, such as greater participation in outdoor activities among males as well as sex-based variation in healthcare seeking behaviors and testing patterns. Our study was not designed to test the effect of these factors on the trends we observed, and they may play a role at the larger population level. However, although smaller in size, our prospective study design can address some of the standardization and timing limitations discussed in their report. In our study, all patients had a physician-confirmed EM. While possible that a small percentage were not infected with *B. burgdorferi* and had alternate explanations for their lesions, we have no reason to think any misclassification would be sex specific. Second, in our sample there were no differences by sex in estimated days of LD duration at initial enrollment and testing. Therefore, we assume that the effect of any diagnostic delays on the evolution of the antibody response would be relatively equally distributed as well. Additionally, patients were tested at standardized intervals in relation to the timing of their diagnosis and treatment, further limiting the effects of any variation in sex-based testing patterns on serologic reactivity. For these reasons, we believe our study suggests that the effects of socio-behavioral differences are less relevant than potential biological factors, at least in the context of our study sample.

The role of menopausal status in clinical presentation or immunological response to *B. burgdorferi* is almost completely unexplored. To our knowledge, only one previous set of studies from Sweden have considered menopausal status, finding that post-menopausal women are most susceptible to re-infection with LD [35, 36]. However, this is likely to be an intriguing area for future study. Levels of the sex-linked hormones estrogen, progesterone, and androgen affect immune cell populations in many ways [12] and menopause is hypothesized to play a role in differential response to certain infections, including SARS-CoV-2 [37, 38]. In our study, females appeared to stratify based on menopausal status for some of the clinical factors that we tested. In each instance, post-menopausal females trended more closely with male patients than the pre-menopausal females did, suggesting an estrogen effect on clinical response. Moreover, within overall and sex-stratified samples, with the exception of LFT, age was not independently significantly associated with specific clinical factors, nor did adjusting for it attenuate the relationships we observed between clinical factors and menopausal status. Therefore, it appears that these relationships are not solely a result of age-related effects.

Conversely, we did not find prominent differences in the overall number or the type of acute symptoms reported by menopausal status or sex. This was particularly evident for many of the most frequently reported early LD symptoms, including fever, myalgia, arthralgia, headache, and fatigue. The differences that we observed by sex were more subtle and were found in symptoms less commonly considered to be present in the diagnosis of early LD, such as gastrointestinal effects (e.g. vomiting, nausea), heart palpitations, photophobia, neck pain, irritability, and sleep difficulty. Apart from the latter two symptoms, each were reported by a higher proportion of females compared to males. Future studies would be needed to replicate the symptoms and the directionality of this finding.

Importantly, future studies should also examine how our findings may impact diagnosis. In particular, current testing algorithms for positivity require a certain number of bands to be reactive. Variation in reactivity could prompt consideration of sex-specific precision in test interpretation or argue for the development of novel diagnostic methods. Altogether, we found a trend toward less severe disease, lower antibody positivity, and more non-traditional acute symptoms among (particularly pre-menopausal) females. Based on these findings, we would hypothesize that misdiagnosis or the lack of specific diagnosis may be more common in this demographic group. Indeed, in unpublished data among all patients enrolled in the *SLICE* cohort with these data collected, 9/47 (19.2%) of pre-menopausal females, 7/58 (12.1%) of post-menopausal females, and 7/113 (6.2%) of males were initially given an alternate, non-LD diagnosis for their signs and symptoms, which is statistically significantly different by group (*p* = 0.048). It will be important to understand how these trends may translate to the population level. Critically, it will also be important to understand if they affect timely, accurate diagnosis and subsequent development of PTLD, for which diagnostic delays and potential exposure to initial non-recommended treatments are considered to increase risk [39, 40]. 

Our study is limited by the geographic specificity of our sample to the Mid-Atlantic US, and it will be important to conduct future studies among patients in other regions. While we attempted to control for potential confounders, there could be others for which we did not gather data or that we otherwise did not think to include which are differentially distributed by sex and could impact our results. In addition, while our overall sample size was relatively large, once stratified by sex and menopausal status, the smaller number of pre-menopausal females in particular may have affected our analyses. The BIF values (ranging from 58.3% to 79.3%) suggest moderate to robust stability of the observed effects, indicating that while there is some sensitivity to sampling variation, the associations between sex, menopausal status, and outcomes remain consistent across the majority of bootstrap resamples. Finally, a modified two-tier test (MTTT) which uses a second immunoassay in place of the traditional Western blot, was cleared by the FDA in 2019 and is increasingly being ordered by physicians and utilized by laboratories [41]. The serologic tests examined in our study represent the STTT approach, whose use may be increasingly eclipsed by the MTTT in the coming years. The relevance of our findings to MTTT will need to be examined as well.

### Perspectives and significance

Our study adds to the little that is known about sex or menopausal status-based differences in LD and suggests that they may be worthy of further characterization and mechanistic exploration. Specifically, future studies should explore variability in the early immune response to infection with *B. burgdorferi*, particularly focusing on associations with estrogen and other sex hormones. Furthermore, as female sex appears to be a risk factor for PTLD, such studies carried out longitudinally in the context of symptom resolution or prolongation may shed light on this and other infection-associated chronic illnesses.

## Conclusions

Our study highlights what little is known about sex-based effects on the clinical presentation and underlying immunology of early LD. In the immediate acute and convalescent period, males in our study had a clinical and laboratory presentation with more apparent objective abnormalities, and we hypothesize this could be due to differences in early host-pathogen interactions. Future studies will be needed to explore these questions and to understand their practical impact on timely diagnosis and treatment.

## Supplementary Information

Below is the link to the electronic supplementary material.

Supplementary Material 1

## Data Availability

The datasets used and/or analyzed during the current study are available from the corresponding author on reasonable request and pending the necessary regulatory approvals.
