# Supplementary material for: Sex and menopause-based differences in presentation of early Lyme disease: A prospective cohort study
Source: Clin Exp Med. 2026 Feb 7;26(1):139. doi: 10.1007/s10238-026-02063-0 (PMC12886233; doi:10.1007/s10238-026-02063-0)
Supplement: Supplementary file 1 — Supplementary Material 1 [file 10238_2026_2063_MOESM1_ESM.docx]

**Supplemental Tables**

**Table S1.** Frequency distribution of the disease composite score (range 0–6) for the total study population and stratified by sex and menopausal status.

| Disease composite  score | Overall  (N=222) | Premenopausal Female  (N=37) | Postmenopausal Female  (N=71) | Male  (N=114) |
| --- | --- | --- | --- | --- |
| 0 | 41 (18.5%) | 10 (27.0%) | 16 (22.5%) | 15 (13.2%) |
| 1 | 42 (18.9%) | 9 (24.3%) | 12 (16.9%) | 21 (18.4%) |
| 2 | 32 (14.4%) | 6 (16.2%) | 12 (16.9%) | 14 (12.3%) |
| 3 | 36 (16.2%) | 4 (10.8%) | 10 (14.1%) | 22 (19.3%) |
| 4 | 33 (14.9%) | 4 (10.8%) | 9 (12.7%) | 20 (17.5%) |
| 5 | 30 (13.5%) | 4 (10.8%) | 9 (12.7%) | 17 (14.9%) |
| 6 | 8 (3.6%) | 0 (0.0%) | 3 (4.2%) | 5 (4.4%) |

**Table S2.** Unadjusted and adjusted logistic regression models examining the association between serologic positivity and sex/menopausal status. Age, duration of illness, systemic steroid use, and thyroid disease were all also considered as potential confounders but determined in prior unadjusted models to be not significant (results in Table 2).

**A.**

| **Model 1**  *Reference pre-menopausal female* | **Unadjusted** | | **Adjusted** | |
| --- | --- | --- | --- | --- |
|  | **OR [95% CI]** | **p-value** | **OR [95% CI]** | **p-value** |
| Post-menopausal Female vs.  **Pre-menopausal Female** | 2.05 [0.85 ,4.97] | 0.149 | 2.08 [0.85 ,5.08] | 0.107 |
| Male vs.  **Pre-menopausal Female** | 3.16 [1.38 ,7.27] | 0.028 | 2.93 [1.26 ,6.79] | 0.012 |
| V2 Test: IgG Only vs. IgM Accepted | 0.54 [0.32 ,0.91] | 0.044 | 0.58 [0.34 ,1.01] | 0.052 |

**B.**

| **Model 2**  *Reference post-menopausal female* | **Unadjusted** | | **Adjusted** | |
| --- | --- | --- | --- | --- |
|  | **OR [95% CI]** | **p-value** | **OR [95% CI]** | **p-value** |
| Pre-menopausal Female vs.  **Post-menopausal Female** | 0.49 (0.20 ,1.18) | 0.149 | 0.48 (0.20 ,1.17) | 0.107 |
| Male vs.  **Post-menopausal Female** | 1.54 (0.85 ,2.79) | 0.150 | 1.41 (0.77 ,2.57) | 0.266 |
| V2 Test: IgG Only vs. IgM Accepted | 0.54 (0.32 ,0.91) | 0.044 | 0.58 (0.34 ,1.01) | 0.052 |

**Table S3.** Ordinal logistic regression models examining the association between composite illness score and sex/menopausal status. We evaluated for differences in all pairwise sex/menopausal groups (Models 1-4). Models comparing male and female groups were adjusted for age and illness duration, whereas only illness duration was adjusted for in the comparison between post- and pre-menopausal females due to collinearity between age and menopausal status and challenges in interpretation. Age and duration of illness were not statistically significant predictors in the final adjusted models.

|  |  | **Odds Ratio [95% CI]** | **p-value** |
| --- | --- | --- | --- |
| **Model 1** *Reference: Female* | Male vs. **Female** | 1.94 [1.20,3.15] | 0.028 |
| **Model 2** *Reference:*  *Pre-menopausal Female* | Post-menopausal Female vs.  **Pre-menopausal Female** | 1.52 [0.76,3.08] | 0.241 |
| **Model 3** *Reference:*  *Pre-menopausal Female* | Male vs.  **Pre-menopausal Female** | 2.26 [1.13,4.58] | 0.044 |
| **Model 4** *Reference:*  *Post-menopausal Female* | Male vs.  **Post-menopausal Female** | 1.68 [0.93,3.04] | 0.118 |
